# The Cost-Effectiveness Analysis of Transplant-Ineligible Myeloma Patients with Bortezomib plus Thalidomide plus Dexamethasone (VTD) or Bortezomib plus Melphalan plus Prednisolone (VMP) Treatment in Southern Taiwan

**DOI:** 10.3390/jpm12020130

**Published:** 2022-01-19

**Authors:** Jeng-Shiun Du, Yi-Chun Kuo, Hon-Yi Shi, Ming-Chung Wang, Li-Ying Wang, Tzer-Ming Chuang, Ya-Lun Ke, Tsung-Jang Yeh, Yu-Ching Gau, Hui-Ching Wang, Shih-Feng Cho, Samuel Yien Hsiao, Yi-Chang Liu, Chin-Mu Hsu, Hui-Hua Hsiao

**Affiliations:** 1 Division of Hematology and Oncology, Department of Internal Medicine, Kaohsiung Medical University Hospital, Kaohsiung 807, Taiwan; ashiun@gmail.com (J.-S.D.); benjer6@gmail.com (T.-M.C.); a9601082@gmail.com (Y.-L.K.); aw7719@gmail.com (T.-J.Y.); cheesecaketwin@gmail.com (Y.-C.G.); joellewang66@gmail.com (H.-C.W.); sifong96@gmail.com (S.-F.C.); ycliu@cc.kmu.edu.tw (Y.-C.L.); e12013@gmail.com (C.-M.H.); 2 Graduate Institute of Clinical Medicine, College of Medicine, Kaohsiung Medical University, Kaohsiung 807, Taiwan; 3 Master of Science in Healthcare Administration, Kaohsiung Medical University, Kaohsiung 807, Taiwan; yichunkuo326@gmail.com; 4 Department of Healthcare Administration and Medical Informatics, Kaohsiung Medical University, Kaohsiung 807, Taiwan; hshi@kmu.edu.tw; 5 Division of Hematology-Oncology, Department of Internal Medicine, Kaohsiung Chang Gung Memorial Hospital, Kaohsiung 807, Taiwan; wangmt@cgmh.org.tw (M.-C.W.); chanel@cgmh.org.tw (L.-Y.W.); 6 Faculty of Medicine, Kaohsiung Medical University, Kaohsiung 807, Taiwan; 7 Department of Biology, University of Rutgers-Camden, Camden, NJ 08102, USA; ucdsacnyu@gmail.com; 8 Cancer Center, Kaohsiung Medical University Hospital, Kaohsiung 807, Taiwan; 9 Center for Cancer Research, Kaohsiung Medical University, Kaohsiung 807, Taiwan

**Keywords:** myeloma, cost-effectiveness, bortezomib

## Abstract

Background: This study aimed to evaluate the cost-effectiveness of treating transplant-ineligible myeloma patients with either a bortezomib plus thalidomide plus dexamethasone (VTD) or a bortezomib plus melphalan plus prednisolone (VMP) treatment in Taiwan. Methods: Newly diagnosed, transplant-ineligible myeloma patients with VTD or VMP therapy were enrolled from two medical centers in southern Taiwan. Quality-adjusted life years (QALYs) were used as the measurement unit of the effectiveness evaluation, and the incremental cost-effectiveness ratio (ICER) was used for comparison between the two groups. A net monetary benefit approach and cost-effectiveness acceptability curve were also used for the cost-effectiveness assessment. A one-way sensitivity analysis was used to check the impact of different parameters. In total, 77 patients were enrolled in the study with 43 patients in the VTD group and 34 patients in the VMP group. Clinical presentations were similar without significant difference, except the VTD group had a higher survival rate (*p* = 0.029). Comparisons of the two groups over an eight-month time horizon revealed a significant lower mean of direct medical costs in the VTD group than in the VMP group (*p* < 0.001), and a significantly higher average QALY was gained (*p* < 0.001). Conclusions: The study demonstrated the greater clinical benefit and cost-effectiveness of VTD compared to VMP therapy in transplant-ineligible, newly diagnosed myeloma patients.

## 1. Introduction

Multiple myeloma (MM) is a malignant B-cell disorder arising from plasma cells and accumulating in the bone marrow; it is the second most common hematological malignancy, accounting for 13% of all hematologic malignancies and 1% of all cancers in the world [1,2,3]. The number of MM patients is expected to increase as a result of a growing elderly population with a median survival of three to five years in recent years [4,5]. MM is not a curable disease, but can be treated with supportive medicines and combination chemotherapies with or without autologous stem cell transplants [6]. With the introduction of novel agents, improvement has been shown in the duration of survival and quality of life [7].

The availability of novel agents currently used by hematologists and oncologists, including proteasome inhibitors, immunomodulatory drugs, and monoclonal antibodies has improved the outcomes of newly diagnosed and relapsed/refractory MM patients significantly. These innovative agents/therapies have also resulted in increased costs and economic burdens to healthcare providers [8,9,10,11]. In addition to clinical effectiveness, cost-effectiveness in patient care is also an emerging issue regarding myeloma patients. Therefore, economic evaluations have become an important tool in helping policymakers allocate resources for public health in many countries [10,12]. However, there is less information on the cost-effectiveness of myeloma therapy in Taiwan.

Though high-dose chemotherapy followed by autologous transplant after induction therapy is recommended for myeloma patients, many of the patients are ineligible for transplant due to old age and/or comorbidities [5,7,13]. Thus, induction therapy with maintained treatment and with supportive medication is the primary treatment option in these patients [6]. In Taiwan, regimens combine the novel agents bortezomib (velcade) with thalidomide and dexamethasone (VTD) or with melphalan and prednisolone (VMP) making them the main protocols for transplant-ineligible patients. New drugs have recently improved the survival rate of patients but also increased the consumption of medical resources. Therefore, this study enrolled transplant-ineligible patients with VTD or VMP therapy from two medical centers in order to assess the clinical benefit and cost-effectiveness under the Taiwanese reimbursement policy. In the study, we applied a standard cost-effectiveness analysis (CEA) methodology that compared the medical costs and health consequences by different regimens [12,14]. The quality-adjusted life year (QALY), which was used as the measurement unit of effectiveness evaluation in this study and is the sum of utility times life year (LY). The cost-effectiveness measure compares the net difference in life expectancy with the net difference in health care cost to obtain a cost per year of life saved [14]. In this way, the overall value of a treatment in terms of incremental costs over incremental health benefit were compared in order to better inform health policy decision making [15]. Therefore, the aim of this study was to assess the cost-effectiveness of either the VTD or VMP treatment over a period of eight months for newly diagnosed myeloma patients who were ineligible for transplant.

The purpose of this study was to investigate the cost–utility of two common drug combinations for MM (VTD and VMP) as a basis for evaluation in future policy revisions on the use of drug therapy for MM patients, so as to formulate a timely and patient-centered regulation with limited medical resources and reduce unnecessary waste and social cost. The results of the study can provide a clinical reference for the selection of drug combinations for the treatment of MM patients in the future, and the cost-effectiveness of the treatment can be included as a reference indicator in addition to disease control and also provide suggestions to health care providers and health authorities for policy planning, improving the overall allocation and utilization of medical resources and reducing unnecessary waste and social cost, thereby enhancing the overall quality of health care.

## 2. Materials and Methods

### 2.1. Sources of Data

**Figure 1:**
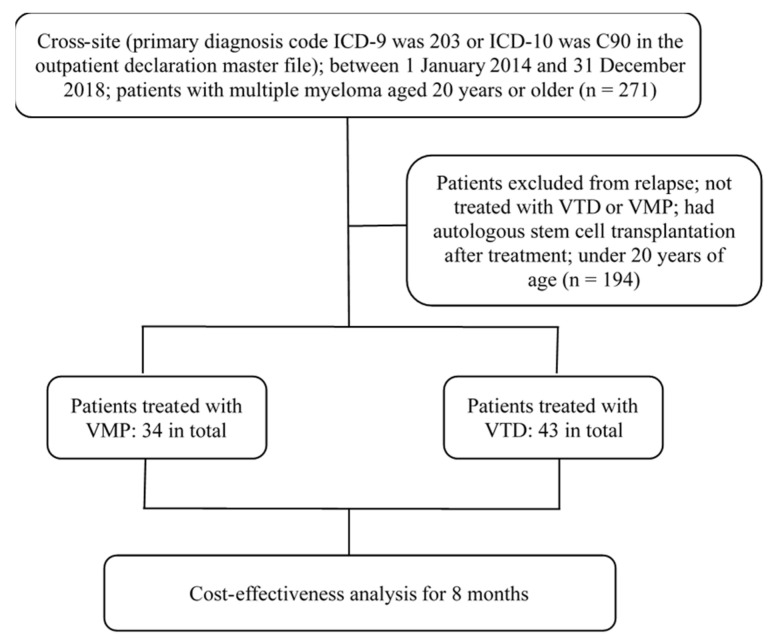
The flow chart of patient enrollment.

Patient files from hospital applications to the Taiwan’s Bureau of National Health Insurance were collected from Kaohsiung Medical University Hospital and Kaohsiung Chang-Gung Memorial Hospital retrospectively under the proof of IRB no. KMUHIRB-E(I)-20200012 (Kaohsiung Medical University Hospital) and no. 201902271B0 (Kaohsiung Chang-Gung Memorial Hospital). Patients with the diagnosis code of ICD-9: 203 and ICD-10: C90 from January 2014 to December 2018 were selected. Patients who received VTD or VMP but underwent a stem cell transplant or those who were eligible for high-dose chemotherapy were excluded from the analysis (Figure 1). Only newly diagnosed myeloma patients with VTD or VMP therapy and who did not receive transplants were enrolled in the study.

### 2.2. Estimation of Cost

The Taiwan’s Bureau of National Health Insurance (BNHI) insurance claims data were analyzed to determine the following hospital medical costs: radiology, physical therapy, hospital room, pharmacy, laboratory, special materials, and others. The medical direct costs include fees for total outpatient cost during the eight-month follow-up after diagnosis, total inpatient cost during the eight-month follow-up after diagnosis. All cost inputs were adjusted to USD 2018 and discounted annually by 3%.

### 2.3. Estimation of Utility

To estimate the quality-adjusted life years (QALYs), a cost–utility analysis often uses “utility scores” (health state valuations) anchored by 0 and 1, where 0 indicates death and 1 indicates full health. Due to a lack of utility data in Taiwan, it was estimated from references [16,17,18,19,20]. The cost–utility of myeloma patients with VTD or VMP therapy was estimated via QALYs. To maintain consistency with the QALYs calculation, this study assumed that the resources used by patients during the eight months of follow-up were only for myeloma treatment.

### 2.4. Statistics

The unit of the study analysis was the individual myeloma patients with VTD or VMP therapy. A decision tree analysis and Markov model were conducted in the present study (Supplementary Figure S1). Details of the cost data used in the model and other input data are shown in Table 1; a more detailed description with all data used in the model is provided in Table S1.

The incremental cost–utility ratio (ICUR) was calculated as the ratio of the difference in mean costs per patient to the difference in mean QALY per patient between the VTD and VMP groups. A willingness-to-pay threshold of gross domestic product (GDP) USD 26,263.5 per QALY was used to assess cost-effectiveness. A program is termed dominant when it is both less costly and/or more effective as evidenced by a lower ICUR. To derive a cost-effectiveness acceptability curve, this study performed nonparametric bootstrapping on the incremental costs and effectiveness with 1000 replications and a cost-effectiveness acceptability frontier. To test the robustness of the model, the one-way sensitivity analysis of assumptions made in this model and the importance of uncertain parameters were tested by varying the parameter values. The results will be presented as a tornado diagram. Microsoft Excel software was used for data processing, and the data were analyzed by IBM SPSS version 20 and TreeAgePro 2017. Frequency and percentage were used for the presentation of descriptive variables. Achi-squared test and independent samples t-test were used for inferential statistics.

## 3. Results

In total, the records of 271 patients with eligible coding were collected from medical record files. Among them, only 77 patients were newly diagnosed with VTD/VMP therapy and ineligible for transplant, thus fulfilling the criteria and were enrolled in the study (Figure 1).

### 3.1. Clinical Characteristics

These 77 patients, 43 patients in the VTD group and 34 patients in the VMP group, showed similar clinical characteristics between the two groups (Table 2). Due to the study focus on patients ineligible for transplant, our population was composed mainly of elderly patients (a mean age of 69.21 years ± 9.85 years) with high disease staging, impaired renal function, and a high ECOG status. Notably, patients with VTD therapy had a higher survival benefit than the VMP group during follow up (*p* = 0.029).

### 3.2. Cost-Saving and ICER

From the files of insurance fee, total medical costs, including inpatient medical cost files and outpatient medical cost files, the VTD group was TWD 952,646 ± TWD 435,629 for eight months, which was less than that of VMP group with TWD 1,028,312 ± TWD 387,414 (*p* = 0.434) (Table 2). The incremental costs of VTD compared to VMP was TWD −75,666 (Table 2). The life expectancy in Taiwan was 77.55 years for males and 84.05 years for females, at an overall average of 80.69 years in 2018. Therefore, the average life year (LY) for the VTD group was 14.1 years, while it was 7.2 years for the VMP group in the study (Table 2). Due to a lack of utility data from Taiwan, the utility data were determined to be 0.58–0.81 in the VTD group and 0.58–0.81 in the VMP group according to the references [16,17,18,19,20,21]. Therefore, the QALY gained was 8.60 to 11.42 in the VTD group and 4.18 to 5.83 in the VMP group (Table 3). From these results, the ICER revealed that VTD therapy provided a greater cost-saving benefit than VMP therapy by saving TWD 13,538 to TWD 17,100 for an increase of one QALY.

### 3.3. Cost-Effectiveness Acceptability Curve

**Figure 2:**
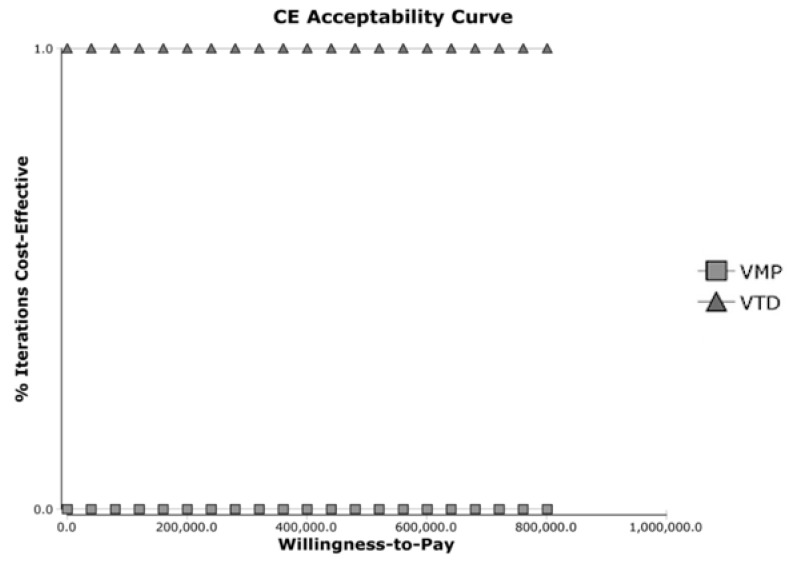
Cost-effectiveness acceptability cure.

In the cost-effectiveness acceptability analysis, the curve favored the VTD group at any price (Figure 2). It revealed that VTD treatment was a more cost-effective choice regardless of willing-to-pay prices.

### 3.4. Net Monetary Benefit (NMB)

**Figure 3:**
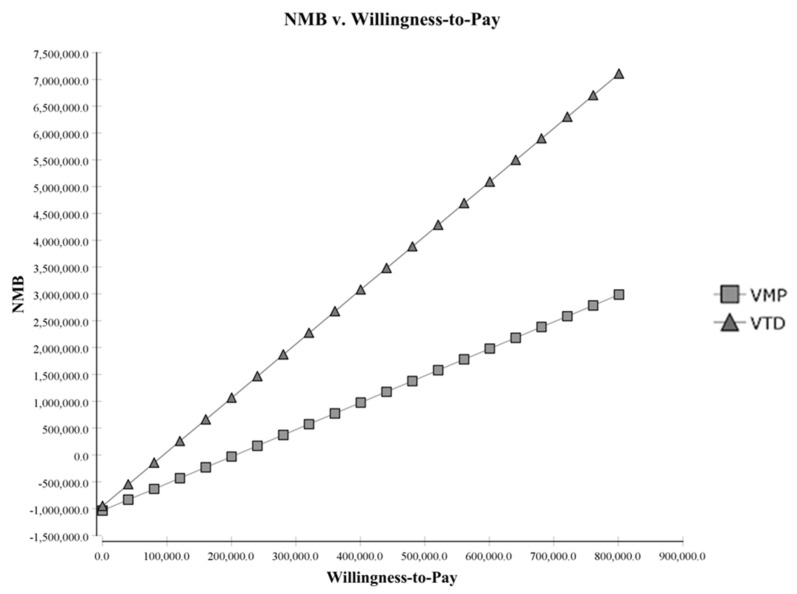
Net monetary benefit with different willing-to-pay prices.

In the net monetary analysis, the VTD group consistently demonstrated an advantage over the VMP group no matter the price of willing-to-pay (Figure 3). Furthermore, the difference in benefit between the two groups increased as the willingness-to-pay went up in favor of VTD therapy.

### 3.5. Scatter Plot

**Figure 4:**
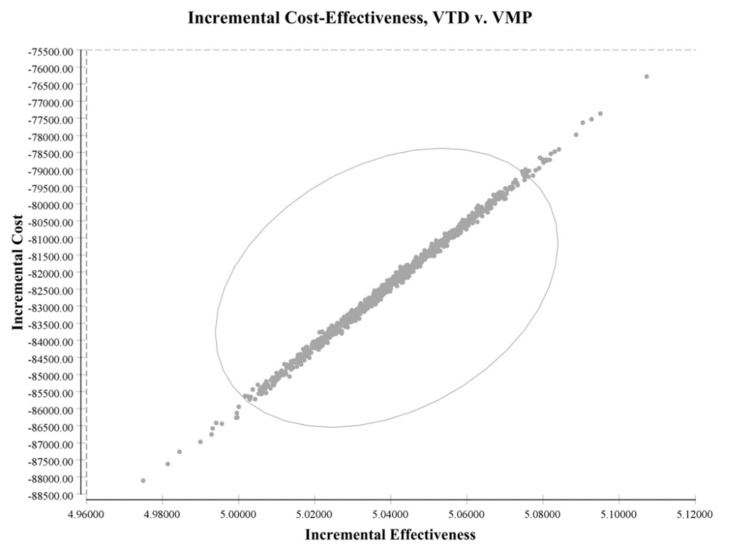
Scatter plot of cost and effectiveness from Monte Carlo simulation.

Previous parameters were used for the Monte Carlo simulation test to identify the incremental cost-effectiveness benefit. After 1000 times of Monte Carlo simulation, with the increase of QALY, the increase of the cost and effectiveness had a positive relationship with a 95% confidence interval (CI) (Figure 4). It revealed the benefit of an incremental cost-effectiveness ratio (ICER) VTD therapy for a saving choice.

### 3.6. One-Way Sensitivity Analysis

**Figure 5:**
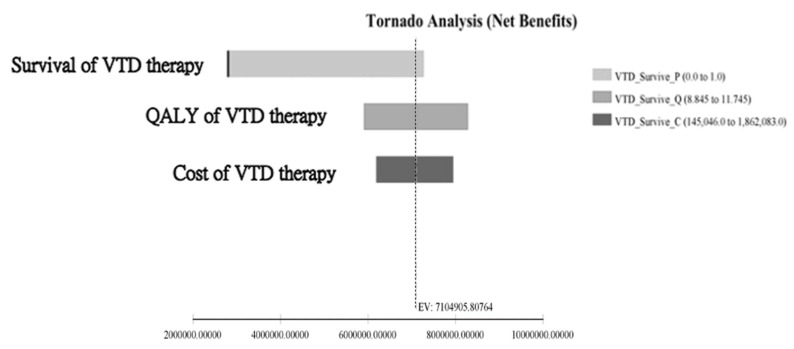
Tornado analysis of the benefit.

A tornado analysis was used for the analysis which accounts for factors affecting the ICER (incremental cost-effectiveness ratio) from the parameters in the VTD and VMP groups. From a single-way sensitivity analysis, the survival rate of VTD therapy, the QALY of VTD therapy, and total medical costs of VTD therapy were the most influential factors for treatment decisions (Figure 5). Among them, the survival of the VTD group had the most important impact on the decision making for VTD therapy.

## 4. Discussion

This is the first cost-effectiveness assessment on newly diagnosed myeloma patients who were transplant-ineligible with VTD and VMP therapy. Bortezomib, the first proteasome inhibitor, approved by the U.S. Food and Drug Administration in 2003, with full approval in 2005, was one of the major components in the combination of myeloma therapy. It had the largest market share of the U.S. from 2000 to 2009 [12]. Since being approved by Taiwan’s Bureau of National Health Insurance, it has been widely used in newly diagnosed myeloma patients in Taiwan with a combination of iMIDs (immune modulators drugs), thalidomide or lenalidomide, and steroid and/or chemotherapy agents. Therefore, we focused primarily on the elderly, transplant-ineligible, newly-diagnosed myeloma patients who received either one of the most common regimens, VTD or VMP treatment, to see the clinical benefit and cost-effectiveness between these two groups.

In the study, we noted the clinical benefit of VTD or VMP therapy with a better survival and less mortality (*p* = 0.029) in the population. Regarding cost-effectiveness, according to files from the Taiwan’s Insurance Bureau of Reimbursement, the VTD group had a higher drug cost than the VMP group by TWD 13,524 per month. However, the total medical costs for the eight-month expense, including inpatient and outpatient costs, favored VTD therapy. Specifically, VTD therapy had a lower total medical expense with an incremental cost of TWD −75,666 compared to VMP therapy. It concurred with previous studies that more effective treatment protocols might decrease the medical needs and medical attention/visits which result in less total medical cost. In this study, the more clinically effective VTD therapy, even with a higher price, was able to maintain a lower total medical cost in the long run. It supports the greater clinical benefit of VTD therapy over VMP therapy with a better outcome and less medical cost, even in the elderly patient population.

The utility value is between 0.38 and 0.69 in myeloma patients with therapies, with a lower value during therapy and a higher level after discharge from the hospital and/or after therapy [8]. It reflected the difference between initial therapy and the improvement of life quality afterward. While it is unfortunate that utility data from Taiwan were incomplete, the utility value of the VTD and VMP groups was able to be derived from the references [8,10,18,22] as was the calculated LY in each group with reference to Taiwanese life expectancy in 2019. Though there was no significant difference in average age, a younger age in the VTD group (63 years vs. 72 years in the VMP group) and a longer survival in the VTD group make the VTD group having more LY than the VMP group, with a difference of 6.9 LY. With a higher survival and a longer LY, the QALY value is 8.6–11.42 in VTD group and 4.18 to 5.83 in the VMP group, with the fact that LY is the major impact factor on QALY. The results revealed the cost-saving benefit of the VTD group had an incremental cost-effectiveness ratio (ICER) of TWD 13,538 to TWD 17,100 with every QALY gained.

The acceptability curve also concurred with the fact that VTD is a better choice at any willingness-to-pay price. In fact, the NMB analysis also supported the fact that at a higher willing-to-pay price, the difference in incremental cost in favor of VTD therapy only became larger. Lastly, a one-way sensitivity analysis revealed that the survival of VTD therapy, the QALY of VTD, and the costs of VTD therapy were the most important factors for ICER among parameters.

Garrison et al. (2013) and Guevara-Cuellar et al. (2016) found that with the incorporation of new drugs into standard treatment regimens, the treatment efficacy of MM patients unsuitable for transplantation has been greatly improved, and the new therapeutic agents were found to be cost effective. Our study demonstrated that the VTD group had a better cost savings than the VMP group. Table 4 presents a summary of the utility values for treatment of MM [23,24]. Clinically, patients with MM who failed to control the disease effectively, the disease deteriorates rapidly and requires more intensive return visits and hospitalization for subsequent management, as was found in this study. The results of the study indicate that VTD was more cost-effective than the VMP group, and the results were consistent. According to the one-way sensitivity analysis, the most important factor affecting the ICER is the survival rate of the VTD group, followed by the quality-adjusted survival of the VTD group. The second most important factor is the quality-adjusted survival of the VTD drug combination, followed by its cost. Clinicians should consider the VTD combination as the first treatment for non-transplant patients with newly diagnosed MM under the current reimbursement of Taiwan’s Bureau of National Health Insurance (NHI).

More novel agents have been developed and introduced for the treatment strategies of myeloma in recent decades, some of which provided improved clinical outcomes. However, the costs of these agents present economic burdens which is also a critical factor in the context of health policy decision making. Therefore, in addition to the clinical benefit, cost-effectiveness assessments of these novel agents are important in the current status [25]. Previous studies showed novel agents, though more expensive, still maintained greater cost-effectiveness for transplant-ineligible myeloma patients as the increase in clinical benefit seemingly outweighed the increased costs [14,23,26]. Our study concurred that the more expensive drug costs of VTD therapy were able to achieve greater cost-effectiveness than VMP therapy by providing both a total cost savings and a greater clinical benefit for transplant-ineligible myeloma patients during eight months of therapy in Taiwan.

The limitations of our study include the samples size and the duration of the follow-up period which limits the conclusion’s application over the long term in a national-wide context. However, the fact that we studied and calculated the data from two different medical centers with no significant difference in clinical characteristics between the two therapeutic groups could partially address these concerns about the study’s generalizability. Secondly, the utility value for the cost-effectiveness analysis is not the exact value from Taiwan as no prior results were available. Hence, our derivation of the utility values could be biased due to different utility values between different countries with different health reimbursement systems. Thirdly, we calculated the medical costs only in these patients; however, some indirect medical costs, such as paramedical needs, were not included. Though the cost-effectiveness of VTD therapy has been shown in our study, difference protocols with treatment settings might give a difference result, as shown in the GEM 2005 trial. Therefore, we should take care of the population and treatment protocol for a clear conclusion [27]. Further studies with more comprehensive and sophisticated settings are warranted for a clearer conclusion [14].

## 5. Conclusions

In conclusion, we studied transplant-ineligible, newly-diagnosed myeloma patients and demonstrated the greater clinical benefit and cost-effectiveness of VTD therapy over VMP therapy with regard to the better survival and cost-savings effects. A further statistical analysis points to the greater survival rate of VTD therapy as a key reason for its higher cost-effectiveness. The result could provide evidence for clinical use and for decisions in a policy setting.

## Figures and Tables

**Table 1:** Key model inputs.

Variable	Value	Range	Distribution	Reference
Possibility				
VTD			Beta	Real Data
Survive	0.9767	0~1		
Death	0.0233	0~1		
VMP				
Survive	0.8824	0~1		
Death	0.1176	0~1		
Cost				
VTD			Gamma	Real Data
Survive	974,976	145,046~1,862,083		
Death	14,804	0~14,804		
VMP				
Survive	1,093,309	0~1,377,610		
Death	597,527	435,491~770,571		
QALYs				
VTD			Gamma	Real Data
Survive	10.2950	8.84500~11.74500		
Death	0.15975	0.13725~0.18225		
VMP				
Survive	5.67950	4.69800~6.56100		
Death	0.06533	0.05452~0.07614		

**Table 2:** Characteristics of patients with VTD and VMP treatment groups.

Patient Characteristics				
Variable	Total (*n* = 77)	VTD (*n* = 43)	VMP (*n* = 34)	*p*-Value
Gender (%)				0.368
Male	42 (54.5)	21 (48.8)	21 (61.8)	
Female	35 (45.5)	22 (51.2)	13 (38.2)	
Age at diagnosis (years)				0.148
65	26 (33.8)	18 (41.9)	8 (23.5)	
≥65	51 (66.2)	25 (58.1)	26 (76.5)	
ECOG performance status (points)				0.764
0	1 (1.4)	0 (0.0)	1 (2.9)	
1	17 (23.3)	10 (25.6)	7 (20.6)	
2	35 (47.9)	19 (48.7)	16 (47.1)	
3	17 (23.3)	8 (20.5)	9 (26.5)	
4	3 (4.1)	2 (5.1)	1 (2.9)	
Disease stage (ISS)				0.589
I	11 (14.7)	5 (12.2)	6 (17.6)	
II	31 (41.3)	19 (46.3)	12 (35.3)	
III	33 (44.0)	17 (41.5)	16 (47.1)	
Bone lesions				0.932
No	13 (18.1)	7 (18.4)	6 (17.6)	
Yes	59 (81.9)	31 (81.6)	28 (82.4)	
Renal function				
Blood urea nitrogen (BUN) mg/dL	31.70 ± 28.6	32.90 ± 33.43	30.03 ± 20.79	0.678
Creatinine mg/dL	2.59 ± 5.57	1.91 ± 2.30	3.24 ± 7.43	0.336
Test values				
Hemoglobin				0.228
<10 g/dL	52 (67.5)	32 (74.4)	20 (58.8)	
≥10 g/dL	25 (32.5)	11 (25.6)	14 (41.2)	
Platelets				0.899
<100,000/mm^3^	12 (15.6)	6 (14.0)	6 (17.6)	
≥100,000/mm^3^	65 (84.4)	37 (86.0)	28 (82.4)	
Calcium				0.903
Free calcium < 5.32 mg/dL or total calcium concentration < 9.9 mg/dL	61 (80.3)	33 (78.6)	28 (82.4)	
Free calcium ≥ 5.32 mg/dL or total calcium concentration ≥ 9.9 mg/dL	15 (19.7)	9 (21.4)	6 (17.6)	
Lactate dehydrogenase (LDH)				0.084
<193 IU/L	42 (60.0)	28 (70.0)	14 (46.7)	
≥193 IU/L	28 (40.0)	12 (30.0)	16 (53.3)	
Albumin g/dL	3.33 ± 0.67	3.29 ± 0.67	3.38 ± 0.68	0.560
Dead or not				0.029
No	52 (67.5)	34 (79.1)	18 (52.9)	
Yes	25 (32.5)	9 (20.9)	16 (47.1)	
Survival (days)	1040 ± 591 (34–2292)	1187 ± 593	853 ± 841	

**Table 3:** Total medical costs, QALY and the ICER (incremental cost-effectiveness ratio) for VTD and VMP therapy.

Variable	Total (*n* = 77)	VTD (*n* = 43)		VMP (*n* = 34)		*p*-Value
	Mean ± SD	Mean ± SD		Mean ± SD		
Within 8 months						
Outpatient costs	830,836 ± 410,554	762,109 ± 359,713		920,389 ± 458,914		0.108
Inpatient costs	154,665 ± 238,072	190,537 ± 282,481		107,923 ± 155,322		0.135
Total cost	958,801 ± 414,411	952,646 ± 435,629		1028,312 ± 387,414		0.434
Incremental costs (VTD-VMP)	−75,666	-		-		
Utility	-	Min: 0.61	Max: 0.81	Min: 0.58	Max: 0.81	
Life year	-	14.1		7.2		
QALY gained	-	Min: 8.60	Max: 11.42	Min: 4.18	Max: 5.83	
ICUR (VTD-VMP)	(−17,100–13,538)	-		-		

**Table 4:** Summary of utility values for the treatment of multiple myeloma (MM).

Authors	Country	Study Design (Population)	Treatment	Results
Garrison et al. 2013 [23]	U.S.	Cost-effectiveness study based on Meta-analysis from RCT (previously untreated, transplant-ineligible patients with MM)	Bortezomib, Melphalan, Prednisone (VMP) vs. Melphalan, Prednisone (MP), vs. Melphalan, Prednisone, Thalidomide(MPT), vs. Lenalidomide, Melphalan, Prednisone with Lenalidomide Maintenance (MPR-R)	Medical costs VMP: USD 119,102. MPT: USD 142,452 MPR-R: USD 248,358. ICER: VMP would confer cost savings and better health outcomes relative to MPT and MPR-R.
Cesar Augusto Guevara-Cuellar, et al. 2016 [24]	Colombia	Cost-effectiveness study based on an RCT (newly diagnosed MM)	Bortezomib, Cyclophosphamide, Dexamethasone vs. Bortezomib, Thalidomide, Dexamethasone vs. Lenalidomide, Dexamethasone	VCD Utility 0.611–0.81 VTD Utility 0.611–0.8 RD Utility 0.5–0.81
Hsiao et al. 2021 [Our study]	Taiwan	Cost-effectiveness study based on retrospective study (previously untreated, transplant-ineligible patients with MM)	Bortezomib, Thalidomide, Dexamethasone (VTD) vs. Bortezomib, Melphalan, Prednisone (VMP)	Medical costs VTD: TWD 952,646 VMP: TWD 1,028,312 QALYs VTD: gained 8.60 to 11.42 VMP: gained 4.18 to 5.83

## Data Availability

The data presented in this study are available from the corresponding author upon reasonable request.

## Supplementary Materials

The following supporting information can be downloaded at: https://www.mdpi.com/article/10.3390/jpm12020130/s1, Figure S1: The decision tree analysis and Markov model; Table S1: The constructive distribution of the cost for each patient in one month (per cycle).
