# Supplementary material for: The Cost-Effectiveness Analysis of Transplant-Ineligible Myeloma Patients with Bortezomib plus Thalidomide plus Dexamethasone (VTD) or Bortezomib plus Melphalan plus Prednisolone (VMP) Treatment in Southern Taiwan
Source: J Pers Med. 2022 Jan 19;12(2):130. doi: 10.3390/jpm12020130 (PMC8880219; doi:10.3390/jpm12020130)
Supplement: Supplementary file 1 [file jpm-12-00130-s001.zip › jpm-1433478-supplementary.pdf]

Supplement Figure S1. The decision tree analysis and Markov model

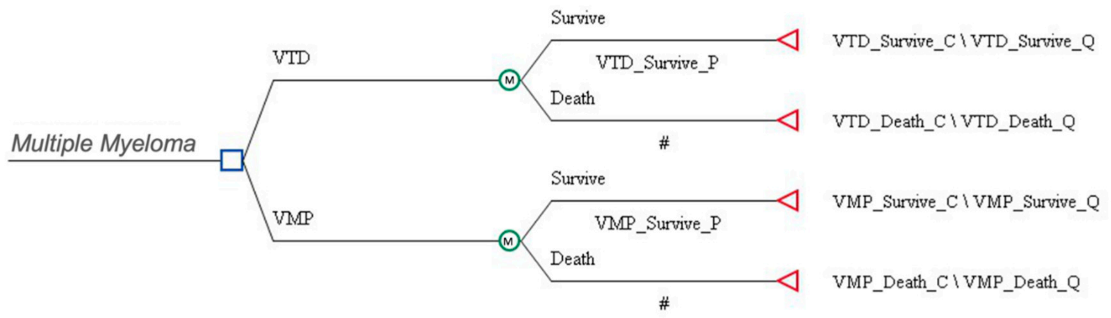

Table S1 The constructive distribution of the cost for each patient in one month (per cycle)

Direct Cost	Outpatient	Diagnostic Fee, Laboratory Fee, Medicine Fee
	Hospitalization	Ward Fee, Diet Fee, Laboratory Fee, X-Ray Fee, Therapeutic Treatment Fee, Rehabilitation Treatment Fee, Blood product fee, Surgical fee, Anesthetic fee, Special Medical Supply Fee, Chemo-Medicine Fee, Medicine Fee, Injection Fee
